# The Preservation of Natural Manures
†The Preservation of Natural Manures. By Alexander McDougal. London: Whittaker. 1856.


**Published:** 1857-01

**Authors:** 


### THE PRESERVATION OF NATURAL MANURES.†

THE valuable fertilising agents of all manures, which require to be preserved, are phosphoric acid and ammonia. The agent by which the author proposes to preserve these valuable substances, is a compound of two acids, the sulphurous and carbonic, and two bases, magnesia and lime. The compound is "a sulphate of magnesia and a carbonate of lime". When mixed with the manure, the sulphurous acid, from its affinity for oxygen, prevents putrescence; while the magnesia enters in combination with the phosphoric acid and the ammonia, forming the triple phosphate of magnesia and ammonia, the best of all compounds for agricultural purposes.

---

† The Preservation of Natural Manures. By ALEXANDER MCDOUGAL. London: Whittaker. 1856.

This disinfectant has, it is said, been extensively used for sanitary purposes. It is supplied to most of the transport ships in the service, and has been used in vaults, coffins, and graveyards with advantage. The expense of it is merely nominal; and it is applicable to the disinfecting and preservation of night soil. Mr. McDougal observes in conclusion :—" There are certain relations established between the animal and vegetable kingdoms, with which all our arrangements ought to harmonise; for unless they do the results will be neither economical nor sanitary. The plant gives off as excrementitious that upon which the animal subsists, while it vegetates luxuriantly upon that which the animal ejects. These two departments of nature are the complements of each other. If animal refuse is carefully preserved in the condition in which it is best adapted to supply food to the plant, it will not only give healthiness to the homestead, but clothe the fields with verdure and endue them with fertility."

